# Primary Solitary Uterine Hydatid Cyst Mimicking an Intramyometrial Cyst: A Case Report

**DOI:** 10.31729/jnma.8536

**Published:** 2024-04-30

**Authors:** Sanyukta Rajbhandary, Kesang Diki Bista, Sunita Bajracharya, Prezma Shrestha, Prerna Mallik

**Affiliations:** 1 Department of Obstetrics and Gynecology, Tribhuvan University Teaching Hospital, Maharajgunj, Kathmandu, Nepal

**Keywords:** *case reports*, *echinococcus*, *hydatid cyst*, *uterus*

## Abstract

Like many agricultural countries, cystic echinococcal zoonotic disease is endemic in Nepal. Incidence of hydatid cyst in liver and lungs are common among the adult population but hydatid cyst of the uterus is an extremely rare entity. We report a case of a 76-year-old menopausal lady who presented with lower abdominal pain for 4 months and underwent laparotomy for provisional diagnosis of myometrial cyst, as shown by MRI scan, however the cyst was found to be primary hydatid cyst of uterus. Postoperatively serological test for hydatid cyst was positive for echinococcus granulosus, further confirmed by histopathological diagnosis. Hence in endemic areas like ours, there should be high index of suspicion of the possibility of hydatid cyst as a differential for cystic pelvic masses.

## INTRODUCTION

Human echinococcosis (hydatid disease) is caused by the larval stages of cestodes (tapeworms) of the genus Echinococcus.^1^ Cystic echinococcal disease is endemic in Nepal, the most affected organ being liver (75%) and the lungs (15%).^2,3^ Isolated hydatidosis of the pelvic cavity is extremely rare, especially involving uterus, documented as an unusual presentation affecting 0.3-0.9% of all hydatidosis cases.^4^ Although the incidence of liver and pulmonary hydatid cyst has been documented in Nepal, there has been no report of uterine echinococcal hydatid cyst in literature. We present a case of uterine hydatid cyst mimicking an intramyometrial cyst in a menopausal lady found incidentally intraoperatively.

## CASE REPORT

**Figure 1.**
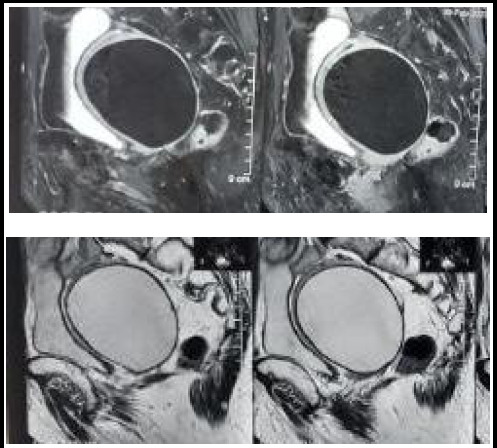
MRI image of the cyst.

A 76 year P4L3 menopausal lady presented with a complaint of lower abdominal pain for 4 months not associated with nausea and vomiting. On abdominal examination, a mass was palpable in the suprapubic region with smooth surface, regular margins and nontender. On per vaginal examination, the uterus was enlarged to 12 weeks size, firm consistency and mobile. On ultrasonography, the uterus measured 10.8 cm × 9.3 cm × 8.7 cm with 252 ml of hypoechoic collection noted in the uterine cavity likely myometrial cyst. MRI revealed a large well defined unilocular cystic lesion in the posterior myometrium 8. 2cm × 6.9 cm × 8.1 cm (volume= 230cc) in subserosal location (Figure 1).

**Figure 2.**
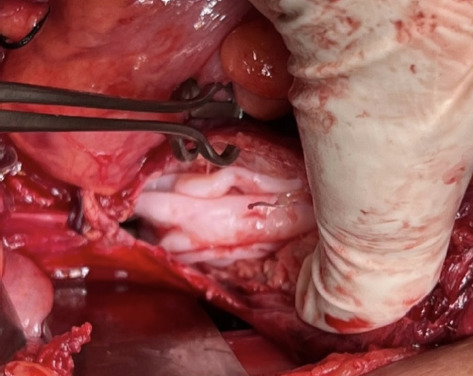
Intraoperative finding of hydatid cyst.

The uterus was found stretched anteriorly with myometrium partially surrounding the cyst suggestive of origin of cyst from the myometrium. A provisional diagnosis of myometrial cyst was made and our patient underwent laparotomy. During laparotomy, the uterus was enlarged to 14 weeks size and adhered laterally to pelvic walls and posteriorly to bowel. Cystic lesion of 10 cm × 10 cm was noted arising from the uterus and adherent to parametrium and bowel posteriorly. Pouch of douglas was obliterated with adhesions and fibrosis. The posterior and lateral wall of the uterus was thinned out due to distension by the cyst. The cyst ruptured during manipulation revealing whitish coloured fluid and pearly white capsule (Figure 2).

**Figure 3.**
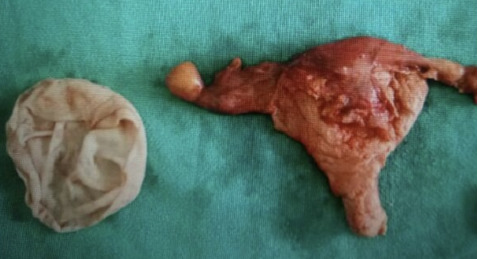
Postoperative specimen of hydatid cyst and uterus with bilateral tubes and ovaries.

Total abdominal hysterectomy with bilateral salpingo-oophorectomy was done (Figure 3). Hypertonic saline solution was instilled in the abdomen for 15 minutes and Inj hydrocortisone 100 mg given iv to prevent anaphylactic shock. Abdomen and pelvis were explored for any other daughter cysts and none were found.

**Figure 4.**
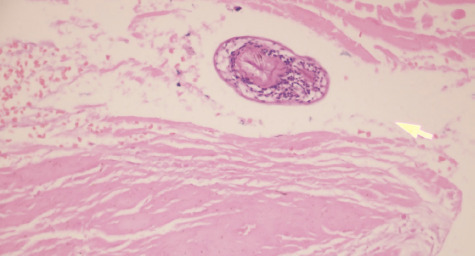
Histopathologic image showing protoscolices of hydatid cyst.

Histopathology confirmed the diagnosis of hydatid cyst of the uterus (Figure 4). Postoperatively echinococcus IgG antibody was sent and was found to be positive. Patient had an uneventful postoperative period and was discharged on tab albendazole 400 mg twice daily for 3 months.

## DISCUSSION

Echinococcal disease is caused by infection due to the tapeworm Echinococcus. Among the four species of Echinococcus causing infection in humans, E. granulosus and E. multilocularis are the most common, causing cystic echinococcosis (CE) and alveolar echinococcosis (AE), respectively. Humans can become infected by ingesting the eggs of parasite that has passed to soil from the feces of canine animals. The eggs of the parasites are hatched by acidic fluid in the stomach. The larvae penetrate the intestinal wall, with the majority entering the portal vein to reach the liver and most of them are captured by the liver. The larvae that escape the hepatic filter reach the lungs and some of them enter the systemic circulation to spread to distant sites such as the brain or abdominal and pelvic organs.^5^

Contamination is most often secondary to intraabdominal rupture of a hydatid cyst of the liver; the daughter vesicles and scolices released settle in the cul-de-sac and continue to develop; secondary endothelialization excludes them from the peritoneal cavity; thus, the intraperitoneal cyst becomes extraperitoneal and appears to be part of the uterine cell tissue. However, primary uterine hydatid cysts have also been reported as in our case.^4^

The cyst formation in the pelvis may remain asymptomatic for long periods of time and may be discovered incidentally or present with compression symptoms. When it is symptomatic, the clinical features depend on the cyst's localization, size and relationship with other organs.

Imaging studies, combined with immunodiagnostic techniques, often help to make a diagnosis. Ultrasonography (USG) is the initial imaging modality of choice followed by Computed tomography (CT) scan and magnetic resonance imaging (MRI) can help diagnose deep-seated lesions and determine the extent and condition of the avascular fluid-filled cysts.^5^

The gold standard test for diagnosis of hydatidosis is the microscopic examination that shows the laminated membrane and scolices.^6^

The rarity of uterine hydatosis, the slow progression of this disease and the heterogeneity of symptoms make the preoperative diagnosis difficult as in our case. Although a preoperative ultrasonography and MRI of abdomen and pelvis was done in our case, only an incidental intraoperative diagnosis of hydatid cyst could be made.

Surgery is the Gold Standard for the treatment of uterine hydatid cysts. Midline laparotomy under the umbilicus remains the best choice. The laparoscopic approach remains controversial because of the lack of experience in treating hydatid cysts by this route, the increased risk of dissemination, and the higher rate of recurrence.^4^

Laparoscopy may be associated with increased risk of spillage due to elevated intraabdominal pressures caused by pneumoperitoneum. The goal of the surgical therapy consists of evacuating the cyst and obliterating the residual cavity. Every effort should be made to avoid fluid spillage, which can lead to secondary seeding of infection and /or anaphylaxis. If spillage does occur, the peritoneum should be washed with hypertonic saline. Postoperatively, the patient should be treated with albendazole for three to six months.^7^

Although hydatid cyst of the uterus is a rare condition, in endemic areas like ours, there should be high index of suspicion of the possibility of hydatid cyst as a differential diagnosis for cystic pelvic masses.
